# Effect of Running Speed on Gait Variability in Individuals with Functional Ankle Instability

**DOI:** 10.3390/e27111131

**Published:** 2025-10-31

**Authors:** Wenhui Mao, Kanglong Zhao, Xiangguo Xu, Mengzi Sun, Kai Wang, Yilin Xu, Li Li

**Affiliations:** 1 School of Sports Science, Nanjing Normal University, Nanjing 210023, China; 12127@njnu.edu.cn (W.M.);; 2 Jiangsu Institute of Sports Science, Nanjing 210013, China; 3 Department of Health Sciences and Kinesiology, Georgia Southern University, Statesboro, GA 30458, USA

**Keywords:** functional ankle instability, postural control, nonlinear analysis

## Abstract

To compare lower limb joint angle variability between functional ankle instability (FAI) and healthy controls (CONs) at different running speeds using linear and nonlinear methods. Fifteen males with right-side FAI and fifteen matched CONs ran on a treadmill at self-selected, 20% faster, and 20% slower speeds. From 25 gait cycles, the mean coefficient of variation (CV), Sample Entropy (SampEn), and largest Lyapunov Exponent (LyE) of hip, knee, and ankle angles were computed. A two-way (two groups × three speeds) mixed-design ANOVA was applied (α = 0.05). No significant interaction effects were observed. No significant differences were observed in the CV. SampEn showed group effects: FAI had lower values in hip horizontal, knee sagittal/coronal, and ankle coronal planes, but higher in the hip sagittal plane. Speed effects showed greater SampEn in the ankle sagittal and lower in the hip coronal plane at slow speed. LyE was reduced in FAI for hip, knee, and ankle sagittal planes. Speed effects indicated higher LyE in the knee sagittal and lower in the hip coronal plane at slow speed. FAI showed reduced variability, particularly in the sagittal plane, reflecting rigid control. Slower speeds increased ankle and knee sagittal variability but decreased hip coronal variability.

## 1. Introduction

Lateral ankle ligament sprain is one of the most common musculoskeletal injuries, affecting 45.1–76.8% of athletes [1]. Over half of athletes with prior sprains experience recurrences, contributing to $4–6 billion in annual U.S. medical costs [2]. Up to 70% of ankle sprain subjects develop chronic ankle instability (CAI), which manifests as either mechanical ankle instability (MAI) or functional ankle instability (FAI) [3]. MAI is characterized by abnormal joint laxity resulting from ligamentous structural laxity or damage, with surgical repair being the primary intervention [4]. FAI is mainly associated with neuromuscular dysfunction [5], which may lead to muscle weakness [6] and impaired balance control [7], and other complications [8], thereby affecting the individual’s motor control capacity [9].

Recent studies have reported that individuals with CAI not only exhibit increased ankle inversion angles [10,11] but also demonstrate altered proximal joint kinematics, such as increased hip adduction [11], elevated hip flexion angles [12], and reduced knee flexion angles [12]. Furthermore, alterations in joint angles were observed not only during gait but also in side-cutting and jump-landing maneuvers [13]. However, joint angle magnitudes alone are insufficient to fully reflect neuromuscular control. Therefore, further analysis of variability is required. Variability refers to the fluctuation of gait parameters across consecutive gait cycles, reflecting the nervous system’s motor control capacity and the musculoskeletal system’s adaptability [14]. In individuals with CAI, altered gait variability serves both as a sensitive marker of injury risk and functional deficits, and as a key reference for rehabilitation and recurrence prevention [15,16,17,18,19,20]. However, conflicting results exist among studies with similar outcome measures [21]. For example, two studies [16,19] collected ankle joint angle variability during running and observed greater variability (calculated via standard deviation, SD) in the frontal plane among CAI individuals, primarily during the early stance phase [16]. This difference is considered a contributing factor to episodes of “giving way” and sensations of instability. However, Kwon et al. [15] observed that during running, CAI individuals exhibited lower joint angle variability in the knee coronal and sagittal planes compared to both healthy controls and copers (defined as those with a history of ankle sprain without developing CAI). Since normal gait should retain a degree of irregularity to accommodate environmental changes [22,23], this suggests that CAI individuals may have a reduced capacity to adapt to task and environmental demands in motor behavior control. Overall, the discrepancies in research results may be attributed to differences in variability calculation methods, gait environments, and other factors, with speed being a key influencing factor [24].

Walking speed has a consistent influence on both kinematic and kinetic parameters of gait in healthy young adults [25]. Increased speed leads to reduced stride time variability [24] and decreased joint angle variability [26]. For example, Blair et al. [26] demonstrated that higher walking speeds resulted in lower lower-limb joint angle coefficient of variation (CV) in healthy young adults, regardless of whether walking occurred on regular or irregular surfaces. However, numerous studies have confirmed [27,28,29] that either a decrease or an increase in walking speed can increase movement variability, exhibiting a U-shaped relationship. Gait stability and the most repetitive gait patterns are optimal when speed approximates the self-selected walking speed. In pathological conditions, speed alters kinematic variability patterns. For instance, Springer et al. [18] observed that increased speed did not alter stride length variability in individuals with CAI, whereas Wanner et al. [16] reported significantly increased ankle frontal-plane variability in CAI athletes at higher speeds. The use of different metrics may be a source of heterogeneity in research outcomes.

In summary, existing studies have investigated lower limb gait kinematic variability in individuals with CAI, yet considerable inconsistencies remain. Furthermore, dynamical systems theory proposes that the human movement system is a complex nonlinear system [23]. The limitation of linear metrics lies in their neglect of the temporal structure embedded within signals. It prevents a conclusive assessment of the dynamic characteristics of the postural control system [30]—a system whose impairment is a hallmark of CAI [7]. Therefore, integrating linear and nonlinear analytical methods can more systematically reveal the mechanisms underlying motor control abnormalities in individuals with CAI [30,31]. Finally, experimental designs focusing solely on the ankle joint have limitations. Given that proximal joint biomechanics are also altered in CAI individuals [32]. Incorporating hip and knee joint analyses may be beneficial. To our knowledge, no study to date has examined the integrated linear and nonlinear variability of the hip, knee, and ankle joints in individuals with FAI across different running speeds.

This study aimed to compare the differences in hip, knee, and ankle joint angle variability (using integrated linear and nonlinear analyses) between individuals with FAI and healthy controls under different running speeds, with the research hypotheses that FAI individuals would exhibit lower joint angle variability and that reduced running speed would increase joint angle variability in FAI individuals.

## 2. Materials and Methods

### 2.1. Participant Recruitment

Fifteen male participants with unilateral FAI on the right side who met all of the following criteria were recruited from the university [33]: (1) a documented history of at least one severe ankle sprain presenting with pain, swelling, and other inflammatory symptoms that resulted in inability to perform normal daily activities for more than one day; (2) Cumberland Ankle Instability Tool (CAIT) score of less than 24 points [34]; (3) two or more episodes of ankle giving way, recurrent sprains, or perceived instability in the affected ankle within the past 12 months; and (4) negative in the anterior drawer test or talar tilt test. They were excluded if they (1) had a history of lower limb fractures or surgical procedures; (2) had acute lower limb injuries, including sprains, within 3 months before the study; (3) were left-leg dominant; (4) had an abnormal arrangement of lower limbs.

Fifteen healthy controls (CONs) were recruited if they met the following criteria: (1) Their right leg was identified as the dominant leg, based on their response to the question: “If you were to kick a ball at a target, which leg would you use?” [35], (2) matched with the FAI group for age, height, body weight, and weekly physical activity time (3) CAIT score >28 points. They were excluded if they had previous lower limb fractures, surgical procedures, or abnormalities in the arrangement of lower limbs.

The study was conducted in accordance with the Declaration of Helsinki and was approved by the relevant biomedical research ethics committee. All participants provided written International Physical Activity Questionnaire (IPAQ) and informed consent before the test.

### 2.2. Experiment Procedure

Prior to formal testing, each participant performed a 3 min warm-up run on the treadmill to adapt to the laboratory environment, become familiar with the procedures, and establish a comfortable running speed. The treadmill speed was adjusted in increments of 0.1 km/h according to participant feedback until a self-selected comfortable speed (preferred speed) was identified. This procedure was repeated three times, and the average was recorded as the participant’s preferred speed. Two additional test speeds were defined relative to this baseline: a faster speed (20% above the preferred speed) and a slower speed (20% below the preferred speed) [36].

**Figure 1:**
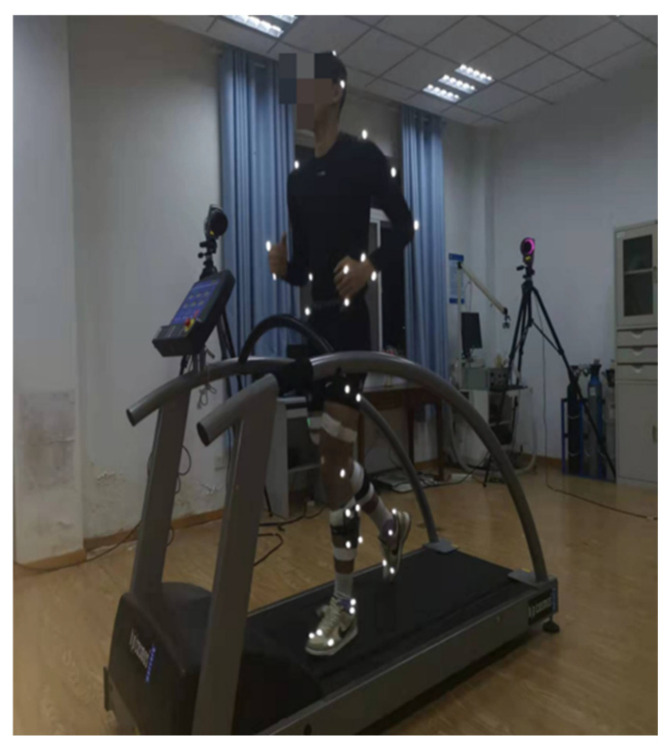
Subjects were formally tested for running.

Each participant then completed three 5 min treadmill runs at the predetermined speeds. The order of speed conditions was randomized using Excel-generated pseudo-random numbers to minimize sequence effects. Participants were instructed to maintain a natural running posture, keep their gaze fixed on the yellow marker, and avoid head movements during the trials, after which the experimenter issued a start signal. Data collection began 30 s after the start signal to allow for adaptation and continued until the completion of the 5 min run. A 5 min rest interval was provided between trials to minimize fatigue. Trials were repeated if interrupted by participant discomfort, external disturbances, marker detachment, or data acquisition failure. For each running condition, 25 consecutive gait cycles were extracted for analysis, starting 30 s after the onset of running. The infrared high-speed optical motion capture system (Arqus500, Qualisys, Gothenburg, Sweden), comprising six cameras, recorded kinematic data at a sampling frequency of 200 Hz (Figure 1).

### 2.3. Data Processing

Gait cycle identification was performed in Qualisys Track Manager, using the peak value of the heel marker along the anterior–posterior axis as the criterion. Subsequently, all participants’ files were imported into Visual-3D (C-Motion, Inc., Rockville, MD, USA) for data processing, where virtual modeling of the trunk and lower limb joints was performed. Following model construction, the data were filtered using a 4th-order Butterworth low-pass filter with a cutoff frequency of 8 Hz. Based on the established model, Euler angles of the lower limb joints were calculated, from which the joint angle data required for this study were extracted and interpolated to 101 points [37].

Previous studies employing the CV have demonstrated that calculating variability for partitioned gait phases can offer additional clinically relevant information. Nevertheless, computing variability across the entire gait cycle remains an efficient and reliable approach, even in studies of analogous neurological conditions [38]. Furthermore, to maintain consistency in data structure with the nonlinear metrics, the CV in this study was calculated based on the entire gait cycle. The CV is calculated as the standard deviation divided by the mean (absolute mean) [39]. The specific formula is as follows:

(1) $${CV}_{Mean\;Joint\;Angles}=mean\left(\frac{SD}{mean}\right)\times 100\%$$

This study adopts the Sample Entropy (SampEn) algorithm proposed by Richman et al. [40] to quantitatively assess the complexity and irregularity of joint kinematics. The SampEn calculation formula is as follows:

(2) $$SampEn\left(m,r\right)=-ln\frac{A\left(m+1,r\right)}{B\left(m,r\right)}$$

Here, m refers to the embedding dimension, which represents the dimensionality of the vectors when reconstructing the time series, and r is the tolerance, typically a multiple of the standard deviation of the time series, used to measure the similarity between two time sequences. The parameter was set to 2, and r was set to 0.2 times the standard deviation [40].

**Figure 2:**
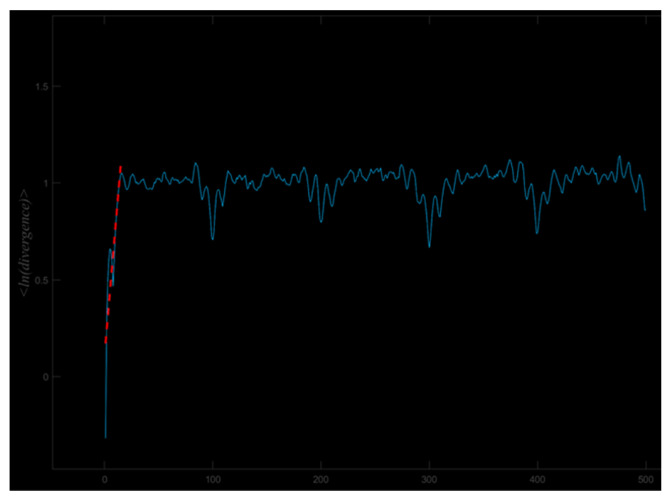
Schematic of the fitting process of the maximum Lyapunov exponent based on the mean logarithmic dispersion.

The largest Lyapunov Exponent (LyE) was calculated using the Rosenstein algorithm to quantify gait dynamic stability by computing the LyE (λ_max_) of lower limb joint angle time series data [41]. The LyE was obtained based on a linear fitting procedure: by performing a linear regression of ln d_i_(k) against K, the slope of the fitted line represents the estimated λ_max_ (Figure 2), where d_i_(k) denotes the distance between neighboring points in phase space at time K, and b is the intercept of the fitted line. The specific formula is as follows:

(3) $$ln{\;d}_{i}(k)\approx \lambda_{max}K+b$$

Based on the formula, custom codes were developed and executed in Matlab (R2023b, MathWorks, Inc., Natick, MA, USA) to calculate the LyE of hip, knee, and ankle joint angles in the sagittal, coronal, and transverse planes [41]. The Matlab function was implemented as: lambda_1 = lyarosenstein (data, m, tau, p, maxiter, fs). Here, lambda_1 denotes the calculated LyE; lyarosenstein is the function implementing Rosenstein’s method; data represents the input time-series of lower limb joint angle signals; m is the embedding dimension for reconstructing the phase space, which was determined using the False Nearest Neighbors (FNNs) algorithm and was found to be 4 for the current dataset; τ is the time delay that defines phase space reconstruction, which was calculated using the Average Mutual Information (AMI) method and was found to be 29; p refers to the mean period of joint angles, derived via Fourier transform; maxiter indicates the maximum iteration count (set to 500) to control computational accuracy; and fs is the sampling frequency (100 Hz). After low-pass filtering at 8 Hz, downsampling from 200 Hz to 100 Hz did not significantly affect the LyE [42].

### 2.4. Statistical Analyses

Statistical analyses were performed using SPSS 27.0 (IBM SPSS Statistics for Windows, Version 27.0., IBM Corp., Armonk, New York, NY, USA). The Shapiro–Wilk test, Levene’s test, and Mauchly’s test of sphericity were first applied to assess data normality, homogeneity of variance, and sphericity, respectively. If the data met the assumptions of normality and homogeneity, a two-way mixed-design ANOVA (version 27) was conducted. In the presence of an interaction effect, simple effect analyses were performed; in the absence of interaction, Bonferroni post hoc tests were applied to examine main effects. All statistical results are reported as Mean ± SD, with α set at 0.05. Effect sizes for ANOVA were evaluated using partial eta squared (η^2^), with thresholds defined as large (η^2^ ≥ 0.14), medium (0.14 > η^2^ ≥ 0.06), and small (0.06 > η^2^ ≥ 0.01) [43]. Cohen’s *d* was interpreted as follows: ≥ 0.80, large; 0.50 to 0.79, moderate; 0.20 to 0.49, small; and < 0.20, trivial [44]. For non-normally distributed or heteroscedastic data, Aligned Rank Transform (ART) ANOVA was employed.

## 3. Results

### 3.1. Participants

A total of 30 participants were recruited, with detailed demographic characteristics presented in Table 1.

### 3.2. Coefficient of Variation

No significant or main effect of group was observed (Table 2). The main effect of speed was significant in the knee joint sagittal plane (F(2,56) = 3.498, *p* = 0.035, partial η^2^ = 0.077). Post hoc comparisons with Bonferroni correction revealed that none of the *p*-values reached the adjusted significance level (*p* > 0.0167).

### 3.3. Sample Entropy

No significant interaction effects were observed (Table 3). The main effect of group was significant in the hip sagittal plane (F(1,28) = 4.515, *p* = 0.037, partial η^2^ = 0.051), hip horizontal plane (F(1,28) = 4.055, *p* = 0.047, partial η^2^ = 0.046), knee sagittal plane (F(1,28) = 7.621, *p* = 0.007, partial η^2^ = 0.083), knee coronal plane (F(1,28) = 5.149, *p* = 0.026, partial η^2^ = 0.058), and ankle coronal plane (F(1,28) = 4.889, *p* = 0.030, partial η^2^ = 0.055).

The main effect of speed was significant in the hip sagittal plane (F(2,56) = 3.140, *p* = 0.048, partial η^2^ = 0.070), post hoc comparisons with Bonferroni correction revealed that none of the *p*-values reached the adjusted significance level (*p* > 0.0167); in the hip coronal plane (F(2,56) = 4.197, *p* = 0.018, partial η^2^ = 0.091), with fast speed greater than slow speed (*p* = 0.005, Cohen’s d = 0.750, 95% CI [0.223, 1.271]); and in the ankle sagittal plane (F(2,56) = 6.645, *p* = 0.002, partial η^2^ = 0.137), with slow speed greater than fast speed (*p* < 0.001, Cohen’s d = −0.938, 95% CI [−1.469, −0.401]).

### 3.4. Largest Lyapunov Exponent

No significant interaction effects were observed (Table 4). The main effect of group was significant in the hip sagittal plane (F(1,28) = 10.669, *p* = 0.002, partial η^2^ = 0.113), knee sagittal plane (F(1,28) = 13.279, *p* < 0.001, partial η^2^ = 0.137), and ankle sagittal plane (F(1,28) = 17.276, *p* < 0.001, partial η^2^ = 0.171).

The main effect of speed was significant in the knee sagittal plane (F(2,56) = 6.432, *p* = 0.003, partial η^2^ = 0.133), with slow speed greater than fast speed (*p* = 0.002, Cohen’s d = −0.829, 95% CI [−1.353, −0.297]); and in the hip coronal plane (F(2,56) = 4.768, *p* = 0.011, partial η^2^ = 0.102), with fast speed greater than slow speed (*p* = 0.007, Cohen’s d = 0.725, 95% CI [0.199, 1.245]).

## 4. Discussion

This study aimed to compare the differences in hip, knee, and ankle joint angle variability (using integrated linear and nonlinear analyses) between individuals with FAI and healthy controls under different running speeds. The observed lower SampEn and smaller LyE in the FAI group partially supported our first hypothesis, while the increased lower-limb sagittal plane variability and decreased hip coronal plane variability resulting from reduced speed partially confirmed the second hypothesis.

### 4.1. Comparison of Variability Between Fai and Con

The results of this study indicated no differences in the CV between the FAI and CON. Hamacher et al. [19] analyzed the coefficient of variation at the ankle joint during running in a CAI population and observed no variability differences in the sagittal plane (plantarflexion/dorsiflexion). However, two studies reported significantly higher variability in the frontal plane (inversion/eversion) of the ankle during running in CAI individuals compared to healthy controls [16,19], particularly during the stance phase, which may explain the recurrent “giving way” and instability sensations in CAI. No CV differences were observed in any joint in our results, which may be attributed to the lack of separation between stance and swing phases in this study, as well as the different coefficient of variation calculation methods used.

FAI individuals demonstrated lower SampEn values across multiple joints and movement planes, indicating more stereotyped movement patterns and reduced adaptability to environmental changes, consistent with previous studies [15,17,18,20]. Specifically, our results showed reduced SampEn in the frontal plane of the ankle joint in FAI individuals, aligning with the results of Terada [20] and Kwon [15]. A potential explanation is that FAI individuals exhibit proprioceptive deficits, leading to insufficient neural input for adapting movement patterns to environmental demands. For example, altered feedback and feedforward neuromuscular control of the peroneus longus muscle may contribute to the loss of optimal gait variability in frontal-plane ankle kinematics [20]. Furthermore, the observed reduction in movement patterns may also represent a pain-avoidance strategy. Beyond the ankle joint, we observed lower SampEn in the knee coronal plane, knee sagittal plane, and hip horizontal plane. This reduction in variability may be detrimental, as insufficient variability can lead to sustained stress on soft tissues (e.g., cartilage, tendons, and ligaments). Repetitive stress may induce pain [45] or even rupture in these tissues. For instance, the decreased SampEn in the knee sagittal plane observed in this study could potentially affect the anterior cruciate ligament (ACL). An epidemiological survey [46] reported that 52–60% of patients with ACL injuries had a history of ankle sprains, further supporting our perspective.

Regarding the LyE, our results indicated that FAI individuals exhibited superior resistance to minor perturbations in the sagittal plane of the hip, knee, and ankle joints. A walking gait study [18] reported that CAI individuals required greater gait disturbances to induce changes, further supporting our results. It is noteworthy that compared to healthy individuals, FAI individuals demonstrated higher SampEn but lower LyE in the hip sagittal plane, which we hypothesize may represent an adaptive outcome at the hip. A potential mechanism emerges: Following an ankle sprain, affected individuals often alter their hip muscle activation patterns to compensate for ankle instability, manifesting as increased co-activation of multiple muscles [47]. This altered muscular strategy may expose the joint angle trajectory to interference from multiple torque vectors. Such interference can lead to unpredictable fluctuations (elevated SampEn) that reflect not intentional flexibility but rather noisy regulation arising from neural adaptation. Concurrently, increased co-activation enhances joint stiffness [48], leading to a dampened response to external perturbations (decreased LyE).

These results suggest that individuals with FAI should avoid abrupt speed changes and refrain from running in environments with high adaptive demands to reduce re-injury risk. Concurrently, anterior cruciate ligament injury prevention strategies may be warranted for this population. Furthermore, the complex compensatory patterns observed at the hip joint may provide valuable insights for understanding the pathophysiology of FAI.

### 4.2. Comparison of Variability Across Speeds

Speed significantly influences gait SampEn and LyE. Nonlinear metrics demonstrated consistent trends in the lower limb sagittal plane: reduced speed led to increased SampEn (ankle joint) and LyE (knee joint). This aligns with previous studies indicating that slower walking speeds elevate variability, as slower locomotion poses greater challenges to motor control [26,49]. For example, Bruijn [50] examined five speed conditions and observed that reduced walking and running speeds were associated with increased LyE in the lower limb sagittal plane, suggesting heightened sensitivity to minor perturbations and reduced local dynamic stability during slow locomotion. Our results indicate that this effect may also exist in running within the sagittal plane. A potential explanation is that running requires substantial propulsive force generation in the sagittal plane. As speed decreases, the utilization of elastic strain energy diminishes, increasing the reliance on active muscle contraction for propulsion [51]. This shift toward greater active neuromuscular adjustment may contribute to elevated SampEn and LyE in the sagittal plane. Additionally, reduced propulsive demand and longer stance phase duration during slow running may further contribute to these changes, though the precise mechanisms remain unclear.

The variability in the hip coronal plane exhibited an opposite trend: decreased running speed led to reduced SampEn and LyE values, indicating more regular hip abduction/adduction movement patterns and enhanced local dynamic stability. A recent study [52] observed a potential negative covariation between coronal and sagittal plane stability, where increased stability in one plane may compromise stability in the other, which might explain the divergent SampEn and LyE patterns observed between the coronal and sagittal planes of the lower limbs.

Additionally, the U-shaped relationship between speed and variability reported in prior literature was not observed in this study for either healthy controls or FAI individuals. Although previous research has identified a U-shaped relationship between speed (including preferred speed and ±20% variations) and the CV of certain spatiotemporal parameters [53], our results indicated no significant differences in joint angle CV. This result is consistent with another study on healthy individuals, which also showed no U-shaped relationship between three running speeds (preferred speed and ±20% variations) and stride interval variability [54]. Therefore, the U-shaped relationship may only manifest in specific metrics under these speed conditions. Our results suggest that a 20% variation threshold might not be optimal for future studies investigating the effect of running speed on joint angle CV.

### 4.3. Limitations

This study has several limitations. First, the analysis was conducted exclusively on young male participants, which may limit the generalizability of the results to female or older populations. Second, the study was constrained by the relatively small number of individuals with moderate or severe ankle instability. Thirdly, this study was conducted on a treadmill, which may have resulted in lower joint angle variability compared to overground running [55]. And last, more data points could provide greater stability for the outcome variables [29]. Future research should consider the influence or interactive effects of factors such as gender and age. Additionally, simultaneous collection of surface electromyography data would help reveal the underlying neuromuscular mechanisms responsible for the observed movement variability.

## 5. Conclusions

FAI individuals demonstrated reduced lower limb variability, particularly in the sagittal plane, suggesting a stable but rigid movement pattern, along with the presence of proximal compensation at the hip joint. Additionally, slower movement speeds led to increased variability in the ankle and knee sagittal plane but decreased variability in the hip coronal plane.

## Figures and Tables

**Table 1:** Participant characteristics.

	FAI Patients (*n* = 15)	Controls (*n* = 15)	*p*
Age (years)	22.00 ± 2.56	23.67 ± 1.05	0.537
Height (cm)	179.00 ± 4.63	176.73 ± 3.51	0.142
Body mass (kg)	72.70 ± 7.76	70.03 ± 4.95	0.271
BMI (kg/m^2^)	22.66 ± 1.88	22.41 ± 1.22	0.671
CAIT (scores)	18.00 ± 4.54	29.33 ± 0.98	0.006 *
IPAQ (MT-min/W)	5796 ± 2361	6212 ± 1124	0.419
Slower speed (km/h)	5.41 ± 0.34	5.51 ± 0.41	0.473
Preferred speed (km/h)	6.77 ± 0.42	6.90 ± 0.51	0.452
Faster speed (km/h)	8.13 ± 0.50	8.29 ± 0.61	0.438

* Significant differences.

**Table 2:** Mean CV results for joint angle at different running speeds and in different groups (%).

Plane	Group	Faster Speed	Preferred Speed	Slower Speed
Hip Sagittal	FAI	0.686 ± 0.363	0.661 ± 0.214	0.854 ± 0.504
	CON	0.690 ± 0.146	0.664 ± 0.186	0.807 ± 0.463
Hip Coronal	FAI	0.680 ± 0.462	0.463 ± 0.121	0.595 ± 0.229
	CON	0.568 ± 0.123	0.536 ± 0.153	0.579 ± 0.140
Hip Horizontal	FAI	1.014 ± 0.541	0.809 ± 0.227	1.091 ± 0.603
	CON	0.805 ± 0.272	0.896 ± 0.340	0.809 ± 0.161
Knee Sagittal **^&,@^**	FAI	1.830 ± 0.604	1.925 ± 0.728	2.592 ± 1.432
	CON	1.840 ± 0.608	1.782 ± 0.546	2.120 ± 0.942
Knee Coronal	FAI	0.440 ± 0.180	0.384 ± 0.114	0.531 ± 0.328
	CON	0.419 ± 0.082	0.422 ± 0.187	0.420 ± 0.133
Knee Horizontal	FAI	0.709 ± 0.258	0.686 ± 0.210	0.948 ± 0.551
	CON	0.741 ± 0.209	0.757 ± 0.261	0.695 ± 0.154
Ankle Sagittal	FAI	0.940 ± 0.446	0.954 ± 0.411	1.179 ± 0.602
	CON	1.087 ± 0.315	0.992 ± 0.319	1.068 ± 0.462
Ankle Coronal	FAI	0.713 ± 0.325	0.695 ± 0.284	0.831 ± 0.305
	CON	0.835 ± 0.433	0.733 ± 0.246	0.774 ± 0.321
Ankle Horizontal	FAI	0.723 ± 0.238	0.763 ± 0.252	0.918 ± 0.356
	CON	0.810 ± 0.243	0.785 ± 0.202	0.818 ± 0.285

**^@^** Significant difference between preferred and slower speeds. **^&^** Significant difference between faster and slower speeds.

**Table 3:** Joint angle SampEn results for different running speeds and groups.

Plane	Group	Faster Speed	Preferred Speed	Slower Speed
Hip Sagittal *^,&^	FAI	0.182 ± 0.024	0.191 ± 0.025	0.210 ± 0.041
	CON	0.175 ± 0.027	0.180 ± 0.032	0.187 ± 0.035
Hip Coronal ^&^	FAI	0.324 ± 0.044	0.310 ± 0.045	0.304 ± 0.043
	CON	0.349 ± 0.036	0.321 ± 0.046	0.306 ± 0.046
Hip Horizontal *	FAI	0.558 ± 0.099	0.588 ± 0.086	0.548 ± 0.118
	CON	0.631 ± 0.094	0.586 ± 0.108	0.604 ± 0.092
Knee Sagittal *	FAI	0.308 ± 0.027	0.311 ± 0.027	0.315 ± 0.023
	CON	0.319 ± 0.023	0.327 ± 0.025	0.334 ± 0.031
Knee Coronal *	FAI	0.438 ± 0.091	0.456 ± 0.100	0.407 ± 0.098
	CON	0.485 ± 0.126	0.497 ± 0.092	0.472 ± 0.124
Knee Horizontal	FAI	0.432 ± 0.061	0.436 ± 0.074	0.437 ± 0.079
	CON	0.440 ± 0.067	0.442 ± 0.068	0.456 ± 0.062
Ankle Sagittal ^&^	FAI	0.281 ± 0.042	0.292 ± 0.049	0.311 ± 0.053
	CON	0.275 ± 0.033	0.297 ± 0.022	0.319 ± 0.025
Ankle Coronal *	FAI	0.354 ± 0.077	0.345 ± 0.075	0.339 ± 0.089
	CON	0.396 ± 0.069	0.386 ± 0.066	0.360 ± 0.064
Ankle Horizontal	FAI	0.474 ± 0.056	0.443 ± 0.080	0.467 ± 0.077
	CON	0.462 ± 0.093	0.452 ± 0.089	0.436 ± 0.083

* Significant difference FAI and CON. ^&^ Significant difference between faster and slower speeds.

**Table 4:** Joint angle largest LyE results for different running speeds and groups.

Plane	Group	Faster Speed	Preferred Speed	Slower Speed
Hip Sagittal *	FAI	5.64 ± 0.81	5.66 ± 1.17	5.98 ± 1.18
	CON	6.17 ± 0.66	6.08 ± 0.54	6.84 ± 0.66
Hip Coronal ^&^	FAI	5.64 ± 1.25	5.72 ± 1.15	4.86 ± 1.14
	CON	6.23 ± 1.10	5.78 ± 0.92	5.30 ± 1.19
Hip Horizontal	FAI	5.48 ± 2.31	5.56 ± 2.17	4.21 ± 1.67
	CON	6.39 ± 1.87	6.07 ± 2.56	4.99 ± 2.09
Knee Sagittal *^,&^	FAI	5.90 ± 0.44	6.34 ± 0.78	6.31 ± 0.80
	CON	6.32 ± 0.63	6.63 ± 0.45	7.09 ± 0.69
Knee Coronal	FAI	5.44 ± 2.38	6.04 ± 1.95	5.22 ± 1.83
	CON	5.62 ± 1.73	5.58 ± 1.87	5.98 ± 1.45
Knee Horizontal	FAI	4.82 ± 1.13	4.91 ± 1.11	4.57 ± 1.40
	CON	5.76 ± 1.52	4.85 ± 1.68	4.65 ± 1.35
Ankle Sagittal *	FAI	5.23 ± 0.56	5.57 ± 0.56	5.68 ± 0.68
	CON	6.02 ± 0.96	5.92 ± 0.68	6.34 ± 0.59
Ankle Coronal	FAI	5.14 ± 1.01	5.29 ± 1.19	5.20 ± 1.42
	CON	4.87 ± 0.88	4.75 ± 1.03	5.47 ± 1.01
Ankle Horizontal	FAI	4.68 ± 1.10	4.83 ± 1.03	4.25 ± 1.29
	CON	5.37 ± 1.69	4.76 ± 1.04	4.67 ± 1.40

^&^ Significant difference between faster and slower speeds. * Significant difference FAI and CON.

## Data Availability

Additional data are unavailable due to privacy and ethical restrictions.
